# A Case Report of COVID-19 With Tracheobronchial Aspergillosis

**DOI:** 10.1155/crdi/4627040

**Published:** 2025-04-13

**Authors:** Haolei Liu, Hailu Jiao, Jun Feng, Hui Gao, Shikui Wu

**Affiliations:** ^1^Department of Respiratory and Critical Care Medicine, The First Affiliated Hospital of Hunan Traditional Chinese Medicine College, Zhuzhou, Hunan, China; ^2^Key Laboratory of Traditional Chinese Medicine for Lung Diseases, The First Affiliated Hospital of Hunan Traditional Chinese Medicine College, Zhuzhou, Hunan, China

## Abstract

The concurrence of COVID-19 and tracheobronchial aspergillosis (TBA) is rarely documented in clinical practice. This report presents a case of TBA in a patient diagnosed with COVID-19 prior to the administration of immunosuppressive agents. This case underscores the necessity of considering fungal infections in patients with COVID-19, even during the early stages of the disease. The combination of timely bronchoscopy, antiviral therapy, and antifungal treatment resulted in favorable therapeutic outcomes for the patient.

## 1. Introduction

TBA is a rare form of invasive pulmonary aspergillosis (IPA) mainly confined to the tracheobronchial tree. The disease presents a range of clinical manifestations, varying from mild cough symptoms to severe conditions such as airway obstruction, which can result in respiratory distress and potentially lead to fatality. TBA typically occurs in immunocompromised individuals, including those with AIDS, hematologic malignancies, solid organ transplants, and those undergoing systemic immunosuppressive therapy [1]. Recent studies conducted during the COVID-19 pandemic have reported increased morbidity and mortality rates among patients co-infected with aspergillosis, thereby complicating the diagnosis and management of COVID-19 [2]. In this paper, we present a case of COVID-19 concurrent with TBA, a combination that has been rarely documented in the existing literature.

## 2. Case Presentation

**Figure 1:**
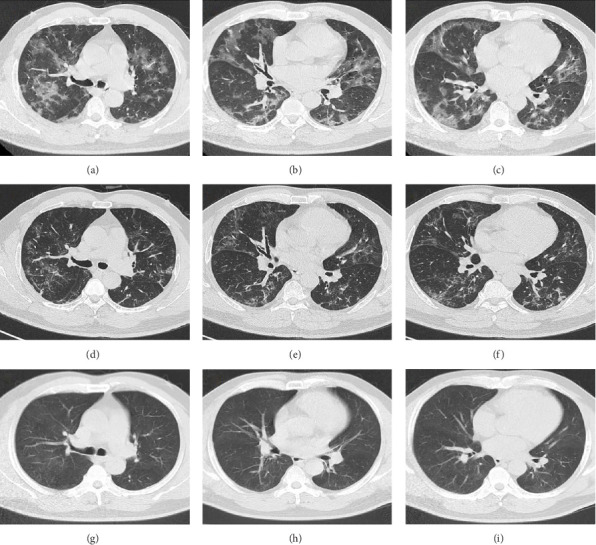
Time course of the patient's chest computed tomography findings (a–c, at admission; d–f, 30 days after admission; g–i, 3 months after admission). The ground glass opacities in both lungs indicate COVID-19 (a–c). The final chest CT showed improvement in lung lesions after treatment (d–f and g–i).

A 54-year-old male patient was admitted to our hospital with a 1-week history of fever, cough, purulent sputum, fatigue, and dizziness. He had a past medical history of hypertension. On admission, his body temperature was 38.3°C, the breath sounds were rough, and wet rales were heard in his lungs. A chest computed tomography (CT) at this time showed diffuse ground glass opacities in both lung (Figures 1(a), 1(b), 1(c)). In addition, laboratory tests showed the absolute values of white blood cell and neutrophils were within the nourmal range, while the absolute values of lymphocytes had decreased to 0.96 × 10^9^/L. His inflammatory markers including C-reactive protein (75.72 mg/L), IL-6 (13.61 pg/mL), and D-dimer (850.00 μg/L) were elevated. Furthermore, routine laboratory evaluations indicated mild hepatorenal dysfunction (aspartate aminotransferase of 70.00 U/L, alanine aminotransferase of 69.00 U/L, and blood urea of 8.14 mmol/L). The other tests including (1–3)-β-D glucan, galaetomannan test and human immunodeficiency virus were negative. Cultures of the patient's sputum were negative for bacteria, fungi, and *Mycobacterium tuberculosis*. Arterial blood gas analysis showed hypoxemia of 69 mmHg.

**Figure 2:**
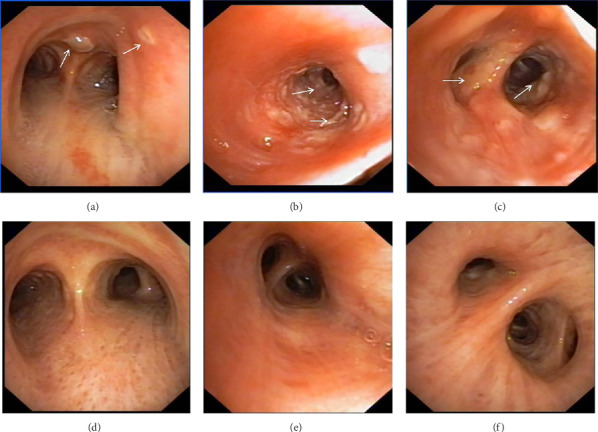
Time course of the patient's bronchoscopy findings (a–c, at admission; d–f, 3 months after admission). The follow-up bronchoscopy procedure confirmed the absence of any bronchial lesions.

**Figure 3:**
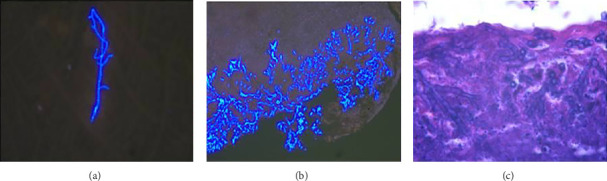
Fungal fluorescent staining in BAL fluid (a) and bronchial mucosa biopsy (b) showed septated hyphae with acute angle branching. Histopathological examination of the bronchial specimen revealed suppurative inflammation and necrosis caused by aspergillus (c). Immunohistochemical results were as follows: P53 (−), EGFR (+), Ki67 (30%+), and special staining: AB (−), PAS (+).

**Figure 4:**
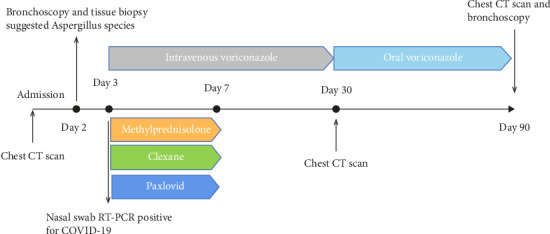
Timeline of the case management interventions.

In want of the identification of the causative pathogen, the bronchoscopy and bronchoalveolar lavage (BAL) on the second day of admission was performed. Bronchoscopy showed congestion, edema of the bronchial mucosa, and multiple white nodular lesions at the trachea, main bronchus, and bronchus intermedius (Figures 2(a), 2(b), 2(c)). Fungal fluorescent staining in BAL fluid and bronchial mucosa biopsy revealed septated hyphae with acute angle branching (Figures 3(a) and 3(b)), which suspected to aspergillus species. Histopathological examination of the bronchial mucosa specimen expressed suppurative inflammation with erosion and necrosis lesion due to aspergillus (Figure 3(c)). However, BAL fluid cultures showed no bacteria, fungi, or tuberculosis, and galactomannan levels were normal. On the third day of admission, His nasopharyngeal swab RT-PCR test was positive for the new coronavirus ORF1ab and N genes. Based on these results, the patitent was diagnosed with COVID-19 and TBA, and treatment with intravenous voriconazole (loading dose of 400 mg every 12 h on days 1 and 2 followed by 300 mg every 12 h), methylprednisolone (40 mg IV daily for 5 days), subcutaneous injection of low molecular weight heparin (Clexane, 4000 IU daily for 5 days), and oral Paxlovid (Nirmatrelvir/Ritonavir, 300 mg of Nirmatrelvir with 100 mg Ritonavir, twice daily for 5 days) was started. As a result, his symptoms, labortatory data (Table 1), and radiographic findings (Figures 1(d), 1(e), 1(f)) gradually improved. Then, the intravenous voriconazole was switched to oral voriconazole (200 mg, twice daily) and the patient was discharged 30 days after admission. Two months after his discharge the patients remains asymtomatic, reexamination of chest CT showed significant lesion absorption in both lungs (Figures 1(g), 1(h), 1(i)), and a repeat bronchoscopy confirmed the absence of any bronchial lesion (Figures 2(d), 2(e), 2(f)). Then the oral voriconazole was discontinued (Figure 4).

## 3. Discussion

This report presents a case of COVID-19 associated with TBA, characterized by non-specific clinical symptoms and chest computed tomography findings, which were subsequently confirmed through bronchoscopic biopsy. The patient received a treatment regimen that included corticosteroid therapy for COVID-19 in conjunction with antifungal medication. After 3 months of treatment, the patient's pulmonary and tracheobronchial lesions were completely resolved.

Aspergillus fungi are common in our environment and can cause aspergillosis in those with weakened immune systems. When inhaled, they may initially infect the tracheobronchial area, resulting in isolated TBA in the early stages. TBA presents with a range of clinical manifestations, including fever, cough, expectoration, wheezing, hemoptysis, chest pain, dyspnea, and rales or stridor upon physical examination. Common findings on CT scans include thickening of the trachea and bronchial walls, nodules distributed along the bronchial trees, atelectasis, bronchiectasis, and patchy consolidation [3]. The bronchoscopic manifestations of TBA have been characterized by mucosal congestion and edema, ulcer, whitish plaques, airway stenosis or obliteration, and cartilage destruction [4]. TBA is often misdiagnosed due to its nonspecific clinical symptoms and imaging findings. Consequently, early bronchoscopy is recommended [5].

The pathogenesis of TBA in COVID-19 patients shares similarities with COVID-19-associated PA (CAPA). The structural damamge of the lung architecture and the treatment of COVID-19 with steroids and immunosuppressants facilitate this coinfection. Furthermore, the virus weakens the immune system and leads to an overexpression of pro-inflammatory cytokines, thereby fostering a highly permissive inflammatory environment that promotes fungal growth [2, 6]. In this case, the patient had not yet received immunosuppressive agents, still developed TBA, highlighting the potential for fungal infections even in the early stages of COVID-19.

The treatment of TBA in COVID-19 patients requires careful consideration of drug interactions, particularly when using voriconazole in combination with ritonavir, a component of Paxlovid. Hence, it is imperative to monitor the blood concentrations of voriconazole. Nebulized liposomal amphotericin B represents a promising alternative, as it delivers targeted therapy to the lesion site while minimizing systemic adverse effects. The duration of antifungal treatment is personalized and typically ranges from 6 to 12 weeks, contingent upon the patient's immune status and treatment response [7]. In cases presenting with central airway obstruction, immediate bronchoscopic intervention is crucial to relieve the obstruction and restore airway patency.

## 4. Conclusions

In conclusion, we report a case of TBA in a patient with COVID-19 before receiving immunosuppressive agent. This case highlights the importance of thinking about fungal infection in patients with COVID-19 even at the early phase. The combination of timely bronchoscopy, antiviral therapy, and antifungal treatment has yielded satisfactory therapeutic outcomes for our patient.

## Figures and Tables

**Table 1:** Changes in patient's inflammatory markers during hospitalization.

	On admission	3 days after admission	7 days after admission	30 days after admission
White blood cell (× 10^9^/L)	4.81	6.81	4.5	4.2
Neutrophils (× 10^9^/L)	3.56	5.42	2.93	2.65
Lymphocytes (× 10^9^/L)	0.96	0.93	1.19	1.26
IL-6 (pg/mL)	13.61	89.52	10.12	5.23
CRP (mg/L)	75.72	135.56	6.2	2.62

## Data Availability

The data that support the findings of this study are available from the corresponding author upon reasonable request.
